# A Comparative Study of Anterior Cruciate Ligament Reconstruction Versus Conservative Treatment

**DOI:** 10.7759/cureus.49148

**Published:** 2023-11-20

**Authors:** Mahesh K Soni, Sharib Shamim, Anil Verma, G K Singh

**Affiliations:** 1 Department of Orthopaedics, Era’s Lucknow Medical College and Hospital, Lucknow, IND

**Keywords:** reconstruction, tegner activity level score, knee injury and osteoarthritis outcome score, international knee documentation committee score, anterior cruciate ligament

## Abstract

Background

The anterior cruciate ligament (ACL) rupture is a common injury with an incidence of 68.6 in 100,000 patients per year. Despite extensive research on ACL rupture, there are insufficient high-quality studies to determine clear treatment strategies for adults lacking the ACL. This study aims to examine the functional differences between surgical and conservative treatment based on the quality of the surgical process.

Methodology

In this prospective, comparative study, a total of 136 patients aged between 18 and 35 years were enrolled per inclusion and exclusion criteria. Using the lottery system, patients were divided into the following two groups: group A (n = 71) and group B (n = 65). Group A was treated with arthroscopic ACL reconstruction and rehabilitation, whereas group B was treated conservatively (rehabilitation). Patient data, including age, sex, body mass index, International Knee Documentation Committee (IKDC) score, Knee Injury and Osteoarthritis Outcome Score (KOOS), Tegner Activity Level (TAL) score, and complications were recorded and compared.

Results

The demographic data were comparable, where males had dominancy in both groups. The mean IKDC and KOOS scores were higher in group A at all follow-ups compared to group B. The scores gradually increased at every follow-up till six months. At the final follow-up, the IKDC and KOOS scores were higher in group A than in group B, and a significant difference was observed among both groups. The TAL score also gradually increased at every follow-up till 6 months. At the final follow-up, the TAL score was higher in group A than in group B, with a significant difference between the two groups (p = 0.0032). Complications in both groups were comparable.

Conclusions

This study showed that both the conservatively treated group and the rebuilt group had identical outcomes, with the exception of the conservative group having greater objectively quantifiable instability. However, at the final follow-up, patients reported feeling just as satisfied with their knee without surgery, demonstrating no subjective difference in activity levels or functional outcomes. Therefore, non-athletes with an ACL-insufficient knee should still choose conservative treatment.

## Introduction

Anterior cruciate ligament (ACL) tears are relatively common, with an incidence of 68.6 per 100,000 patients per year, and tend to affect young, active people [1]. Without the ligament’s restraint, the rotation axis might shift medially, making the lateral tibial plateau more vulnerable to anteroposterior instability [1-4]. Posterior cruciate ligament injuries rarely occur in isolation and have an incidence of 1.8 per 100,000 patients per year. An increased risk of posttraumatic osteoarthritis of the knee, limited knee function with reduced activity level, and lowered quality of life are linked to chronic anteroposterior instability, which persists even after surgical treatment in 8-50% of cases and 75-87% of patients treated conservatively [5,6]. Although there have been numerous studies on ACL tears, there are not enough high-quality studies to provide definitive treatment recommendations for individuals with ACL tears. ACL reconstruction using an autologous tendon graft is recommended for patients who also sustain injuries to their collateral ligaments, meniscal tears amenable to reconstruction, or who report high subjective instability or loading demand [7-9]. It is the first-line treatment to restore passive knee joint stability in symptomatically unstable patients. Evidence has shown that reconstructing the ACL can reduce the risk of further damage to the meniscus and cartilage and allow patients to resume their preinjury physical activity levels [10,11]. This study examined the functional differences between surgical and conservative treatment based on the quality of the surgical process, as the evidence for preferring particular treatment options was limited.

## Materials and methods

This study was conducted at Era’s Lucknow Medical College and Hospital, Lucknow, in the Department of Orthopaedics. The study was a prospective, comparative study of patients with complete ACL tears from 2019 to 2022. All patients with knee injuries who presented to our hospital’s emergency or outpatient department were evaluated for ACL rupture. Ethical clearance (approval number: R. Cell/EC/2019/102) and informed consent were obtained before study initiation. Patients aged between 18 and 35 years of either gender, patients with isolated ACL tears less than four weeks old in a previously uninjured knee, and Tegner Activity Level (TAL) scores of up to 5 were enrolled. Using the lottery system, patients were further divided into the following two groups: group A (n = 71) and group B (n = 65). Group A was treated with arthroscopic ACL reconstruction and rehabilitation, whereas group B was treated conservatively (rehabilitation).

Diagnostic arthroscopy was performed via anteromedial and anterolateral portals positioned adjacent to the lateral and medial patellar tendon borders at the level of the patella’s inferior pole. The arthroscope was inserted through an anterolateral portal, an anteromedial portal was created under arthroscopic guidance, and instruments were introduced through the anteromedial portal. A comprehensive diagnostic arthroscopy was performed in group A. Any intra-articular pathologies were treated at that time, and meniscal procedures were performed. A minimal notchplasty of soft tissue was conducted for visual purposes only. The anatomical site of ACL insertion on the lateral wall of the intercondylar fossa was determined and marked with an AWL or RF.

Graft harvest and preparation

Patients’ semitendinosus and gracilis tendon grafts were harvested through an oblique 3-4 cm incision situated anteriorly and medially on the pes anserinus of the tibia. Incisions were made in the subcutaneous tissue along the incision line, where a thin vein was frequently detected. The pes anserinus insertion was exposed by abrupt dissection, and the gracilis tendon was located by palpating and rolling beneath the sartorius and semitendinosus tendons. Maintaining ample exposure, the semitendinosus tendon was pulled forward with a curved clamp or braided suture, and the dissection was done proximally up the thigh. The semitendinosus tendon was detached from its tibial insertion, and the insertion site was circumscribed and undermined with a periosteal elevator. A double whipstitch of the Krackow type was placed near the insertion, and fibrous extension to the gastrocnemius and semimembranosus muscles was released. The tendon was released by applying controlled tension to the tendon and advancing the extractor proximally. Both tendons were harvested one at a time and prepared and their length was determined to be adequate. The graft was placed under tension and covered with moist saline gauze for 20-30 minutes while the cavity and tunnel preparation continued.

Femoral and tibial tunnel preparation

Creating apertures in the femoral socket was necessary for the arthroscopic evaluation and treatment of related conditions. The notch and inner aspect of the lateral femoral condyle were prepared so that the posterior aspect of the lateral condyle was visible and the ACL impression was clearly visible. The femoral tunnel was drilled through the anteromedial bundle footprint with a 4 mm or 5 mm offset endofemoral aimer, and the knee was flexed to 90 degrees to position the guide. A long 2.4 mm drill-tip guidewire was advanced through the physiometric point on the posterolateral portion of the femoral condyle until it penetrated the lateral femoral cortex. To determine the position of the guidewire, its exit from the cortex was felt beneath the epidermis. The lateral femoral cortex was perforated by advancing a cannulated 4.5 mm endobutton drill bit over the passage pin. To prepare the femoral cavity, the endobutton depth probe was used to measure the total length of the femoral tunnel, and an endoscopic drill bit matching the graft’s diameter was selected. A bead pin with a thread was passed through the epidermis and into the femoral socket, leaving the thread in the femoral canal and knee joint. To ensure that the graft was secured in a physiometric, impingement-free position, the tibial guide was modified according to the tunnel length and direction. To align the grafts and eliminate slackness, the grafts were subjected to 15-20 cycles of complete flexion and extension of the knee. A bioscrew was used to secure the distal end of the graft while maintaining the anteroposterior position of the tibial with regard to the femoral condyles.

All patients were given a self-administered patient questionnaire containing Knee injury and Osteoarthritis Outcome Score (KOOS), International Knee Documentation Committee (IKDC) score, and TAL score after obtaining their informed consent. During surgery, the surgeon completed a questionnaire regarding knee injury history, general and knee clinical examination, radiological examination, and surgical technique. All patients were examined for instability during clinical follow-up, and IKDC, KOOS, and TAL scores were recorded. One orthopedic surgeon maintained all records and evaluated the outcomes.

Statistical analysis

SPSS Version 26.0 software (IBM Corp., Armonk, NY, USA) was used for statistical analysis. Descriptive statistics were presented as mean, standard deviation, frequency, and ratios. Categorical data were analyzed using the chi-square test, and continuous data were analyzed using the Student’s t-test. Significance was evaluated at a p-value <0.05.

## Results

The mean ages in both group A (28.14 ± 5.35) and group B (27.32 ± 6.12) were comparable. Male patients had dominancy in both groups (p = 0.3394). The mean BMI in group B (26.21 ± 3.32) was higher than in group A (25.62 ± 3.21), although insignificant (Table 1).

**Table 1:** Demographic parameters of patients among the groups. SD = standard deviation; t = Student’s t-test; χ^2^ = chi-square test; p = probability value

Demographic parameters		Group A (N = 71)		Group B (N = 65]		P-value
		Mean	SD	Mean	SD	
Age		28.14	5.35	27.32	6.12	t = 0.8335, p = 0.4060
Gender	Male	62	87.32%	60	92.31%	χ^2^ = 0.9127, p = 0.3394
	Female	9	12.68%	5	7.69%	
Body mass index		25.62	3.21	26.21	3.32	t = 1.053, p = 0.2941

The mean IKDC score was higher in group A at all follow-ups than in group B. It gradually increased at every follow-up till the sixth month, as shown in Table 2.

**Table 2:** International Knee Documentation Committee scores of the patients among the groups. IKDC = International Knee Documentation Committee; SD = standard deviation; t = Student’s t-test; p = probability value

IKDC score	Group A (N = 71)		Group B (N = 65)		P-value
	Mean	SD	Mean	SD	
Preoperative	35.23	2.32	25.61	1.53	t = 28.27, p < 0.0001*
6 weeks	50.84	1.96	49.95	2.62	t = 2.255, p = 0.0257*
16 weeks	65.42	1.50	63.58	1.94	t = 6.216, p < 0.0001*
6 months	76.65	2.64	76.52	2.31	t = 0.3044, p = 0.7613
Final follow-up	74.46	2.81	73.68	2.82	t = 1.614, p = 0.1088

**Figure 1:**
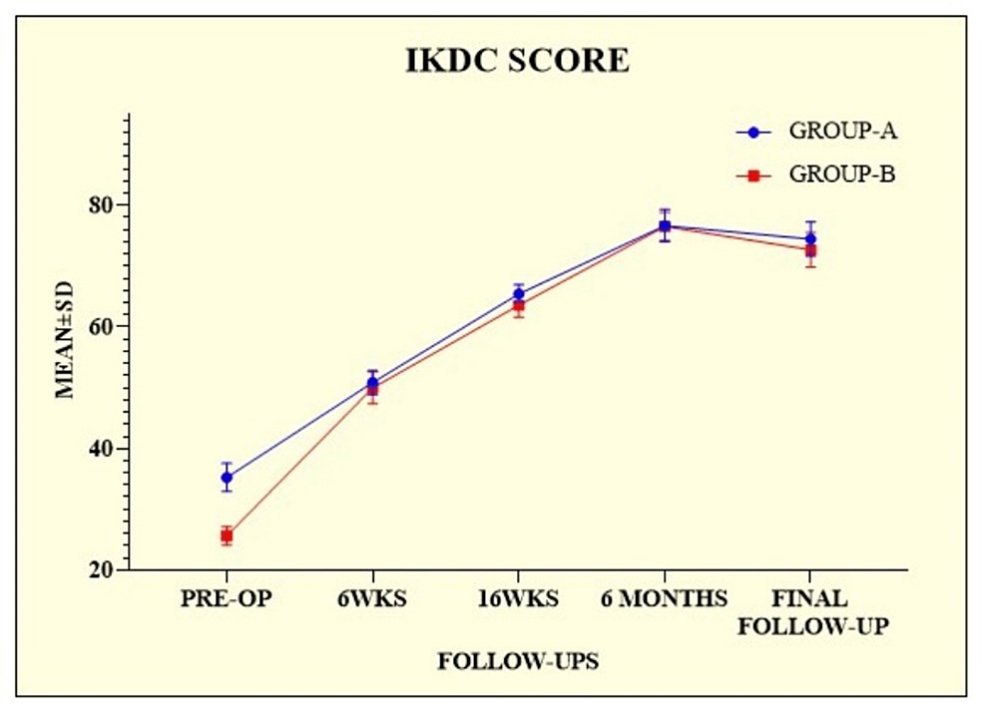
International Knee Documentation Committee scores of the patients among the groups. IKDC = International Knee Documentation Committee; SD = standard deviation; PRE-OP = preoperative; WKS = weeks

The difference between the two groups was significant at every follow-up, except for the sixth month (p = 0.7613) (Figure 1).

The mean KOOS score was substantially higher in group A at every follow-up than in group B, as shown in Table 3.

**Table 3:** Knee Injury and Osteoarthritis Outcome scores of the patients among the groups. KOOS = Knee Injury and Osteoarthritis Outcome Score; SD = standard deviation; t = Student’s t-test; p = probability value

KOOS score	Group A (N = 71)		Group B (N = 65)		P-value
	Mean	SD	Mean	SD	
Preoperative	37.61	1.85	37.42	1.62	t = 0.6347, p = 0.5267
6 weeks	55.58	2.64	53.17	1.48	t = 6.485, p < 0.0001*
16 weeks	68.29	2.13	67.11	1.21	t = 3.924, p = 0.0001*
6 months	81.34	2.42	80.62	3.28	t = 0.4915, p = 0.1453
Final follow-up	76.81	2.65	75.84	3.62	t = 1.793, p = 0.0752

**Figure 2:**
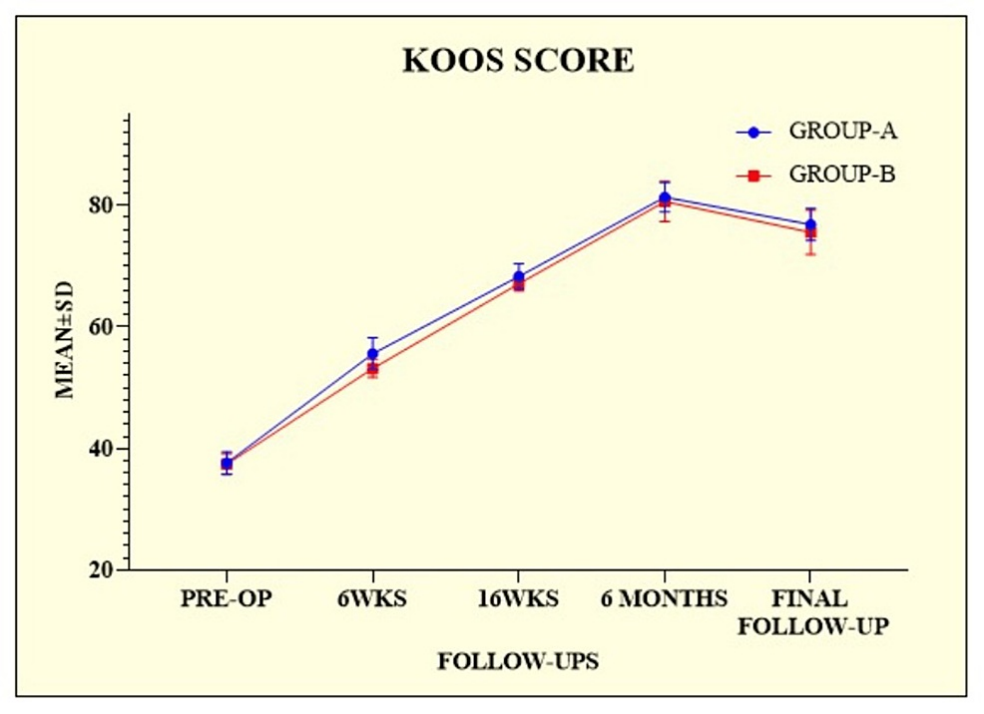
Knee Injury and Osteoarthritis Outcome scores of the patients among the groups. KOOS = Knee Injury and Osteoarthritis Outcome Score; SD = standard deviation; PRE-OP = preoperative; WKS = weeks

During the sixth week, 16th week, and final follow-ups, a significant difference was observed among the groups (Figure 2).

The mean TAL score gradually increased at every follow-up till the sixth month (Table 4).

**Table 4:** Tegner Activity Level scores of the patients among the groups. TAL = Tegner Activity Level score; SD = standard deviation; t = Student’s t-test; p = probability value

TAL score	Group A (N = 71)		Group B (N = 65)		P-value
	Mean	SD	Mean	SD	
Preoperative	0.97	0.26	0.99	0.31	t = 0.4088, p = 0.6833
6 weeks	1.39	0.32	1.26	0.38	t = 2.164, p = 0.0322*
16 weeks	3.45	0.67	3.11	0.42	t = 3.508, p = 0.0006*
6 months	4.39	0.46	4.16	0.47	t = 2.883, p = 0.0046*
Final follow-up	4.11	0.48	3.95	0.53	t = 1.847, p = 0.0669

**Figure 3:**
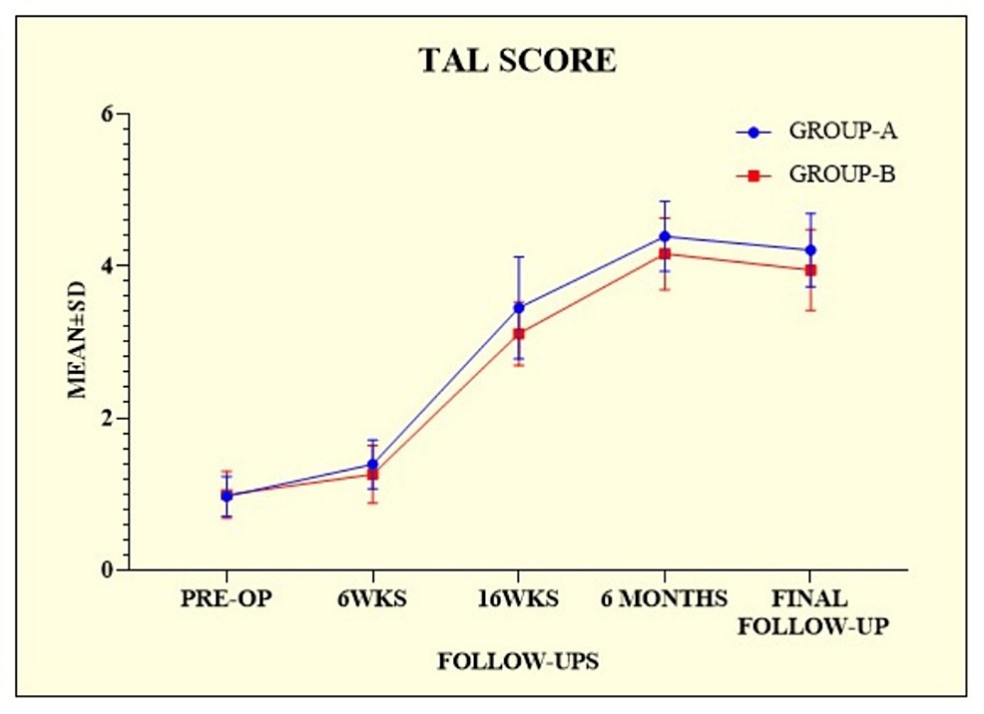
Tegner Activity Level scores of the patients among the groups. TAL = Tegner Activity Level score; SD = standard deviation; PRE-OP = preoperative; WKS = weeks

TAL score showed significance at every follow-up (Figure 3).

In group A, the most common complication was anterior knee pain (2.63%), while in group B hemarthrosis (2.90%) and urinary retention (2.90%) were the complications noted. However, a non-significant difference was noted in the complication among groups (Table 5).

**Table 5:** Complications in the patients among the groups. χ^2^ = chi-square test; p = probability value

Complications	Group A (N = 71)		Group B (N = 65)		P-value
	N	%	N	%	
Anterior knee pain	2	2.63%	0	0.00%	χ^2^ = 6.300, p = 0.2781
Hemarthrosis	1	1.32%	2	2.90%	
Hardware prominence	1	1.32%	0	0.00%	
Superficial infection	1	1.32%	0	0.00%	
Urinary retention	0	0.00%	2	2.90%	
Total complications	5	6.58%	4	5.80%	
None	66	86.84%	61	88.41%	

## Discussion

ACL tears are frequent musculoskeletal injuries. Globally, the number of ACL reconstructions is increasing. It is primarily a sports-related injury that frequently causes knee instability. Significant progress has been made in transforming open surgical procedures into minimally invasive arthroscopic procedures. Treatment should always be individualized for each patient. There is insufficient long-term evidence to recommend one treatment (operative or conservative) over the other. Multiple factors should be considered, including complaints, degree of instability, sports preferences, age, and commitment to a six-month rehabilitation program. ACL reconstruction does not reduce the risk of secondary knee osteoarthritis or restore normal knee kinematics, but it does reduce complaints of knee instability. These complaints about giving way remain the most significant indicator for this operation. We know that the incidence of ACL reconstructions among non-athletes is increasing, but the incidence remains unknown [12,13].

This study was conducted to provide additional insights into the outcomes of ACL injuries in non-athletes. A total of 136 patients were enrolled in this prospective randomized controlled trial. Patients were divided into the following two groups: group A (n = 71) and group B (n = 65). Regarding age and body mass index (BMI), there was no statistically significant difference between the groups (p = 0.4060 and p = 0.2941, respectively). Similarly, Kumar et al. [14] enrolled 132 patients into two groups, 68 in group A and 64 in group B, calculated the mean age and BMI, and observed no statistically significant difference (p = 0.889 and p = 0.115, respectively). Meuffels et al. [15] enrolled 50 patients and divided them into two equal groups of 25 each. The two groups were similar with respect to age, gender, and BMI and showed no significant differences. Male dominance was observed among the groups in our study, but the difference was not statistically significant (p = 0.3394). Similarly, the study by Kumar et al. [14] revealed male dominance with a non-significant difference between the groups (p = 0.299). Their study aimed to provide more information on the outcomes of ACL injuries in non-athletes. Both groups were comparable in terms of age, gender, and level of activity. The operated group performed better at 10 and 16-week postoperative follow-ups (IKDC, KOOS, and TAL were statistically significant). Still, at the final postoperative follow-up, both groups performed similarly (IKDC, KOOS, and TAL were statistically insignificant). These outcomes were compared to previous findings for ACL injuries treated conservatively or through reconstruction [15-18]. Similarly, in our study, at the final follow-up, the IKDC scores for group A were 74.46 ± 2.81, and those for group B were 72.68 ± 2.82, with a significant difference between the groups (p = 0.0003). On comparing the IKDC score to the finding of Kumar et al. [14], we observed a difference in scores; however, the difference was not statistically significant (p = 0.140). In our study, we calculated the mean KOOS score and TAL score. At the final follow-up, the KOOS score was 76.81 ± 2.65 for group A and 75.54 ± 3.62 for group B, with a statistically significant difference between the two groups (p = 0.0203). Alternatively, the TAL scores at the final follow-up were 4.21 ± 0.48 for group A and 3.95 ± 0.53 for group B, with a statistically significant difference between the two groups (p = 0.0032). On comparing the KOOS and TAL scores with the study by Kumar et al. [14], the final KOOS score for group A was 77.75 ± 2.42, and for group B was 76.79 ± 3.0. Group A had a higher TAL score (4.07 ± 0.76) than group B (3.94 ± 0.75). A significant difference was observed between the groups in terms of scores (p = 0.052 and p = 0.304, respectively), which was consistent with our previous findings. Five (6.58%) patients in group A experienced complications, including two (2.63%) with anterior knee pain. Each complication of hemarthrosis, hardware prominence, and superficial infection accounted for one (1.32%) patient. Similarly, four (5.80%) patients in Group B experienced complications. There were two (2.90%) patients with haemarthrosis and two (2.90%) patients with urinary retention. Consequently, a non-significant difference was observed between the two groups (p = 0.2781). In the study by Kumar et al. [14], group A had four patients with anterior knee pain that was alleviated by physiotherapy and oral analgesics, one patient had a superficial infection that was treated with antibiotics, and three patients reported hardware prominence (at tibial post) that necessitated the removal of the tibial post-implant in the sixth month postoperatively. In group B, there were four hemarthrosis cases and three urinary retention cases. Symptomatic treatment alleviated these conditions.

The small sample size, single-center study, and the short-term follow-up are the limitations of this study. We recommended further large multicentric studies with optimal effect sizes for more reliable and generalizable findings.

## Conclusions

This study showed that ACL reconstruction is a successful knee stabilization procedure. ACL reconstruction provides the same sensation and functionality to these patients as conservative treatment. Reconstruction of the ACL is currently regarded as the gold-standard treatment for restoring knee stability and enhancing knee function. However, conservative care is deemed a treatment alternative. In young and athletically active patients, ACL rupture is a severe injury with significant long-term consequences, including functional impairment, posttraumatic osteoarthritis of the knee, and decreased quality of life. There is no subjective difference in activity levels or functional outcomes between those with and without knee surgery at the final follow-up. Therefore, non-athletes with an ACL-deficient knee should pursue conservative treatment options. We found no evidence that one treatment was more dangerous than another. Additional randomized controlled trials and large prospective long-term cohort studies involving surgically and non-surgically treated patients will contribute to our knowledge of this prevalent and disabling condition.

## References

[1] Sanders TL, Maradit Kremers H, Bryan AJ (2016). Incidence of anterior cruciate ligament tears and reconstruction: a 21-year population-based study. Am J Sports Med.

[2] Spindler KP, Wright RW (2008). Clinical practice. Anterior cruciate ligament tear. N Engl J Med.

[3] Seitz H, Chrysopoulos A, Egkher E, Mousavi M (1994). [Long-term results of replacement of the anterior cruciate ligament in comparison with conservative therapy]. Chirurg.

[4] van Yperen DT, Reijman M, van Es EM, Bierma-Zeinstra SM, Meuffels DE (2018). Twenty-year follow-up study comparing operative versus nonoperative treatment of anterior cruciate ligament ruptures in high-level athletes. Am J Sports Med.

[5] Mall NA, Chalmers PN, Moric M, Tanaka MJ, Cole BJ, Bach BR Jr, Paletta GA Jr (2014). Incidence and trends of anterior cruciate ligament reconstruction in the United States. Am J Sports Med.

[6] Frobell RB, Roos HP, Roos EM, Roemer FW, Ranstam J, Lohmander LS (2013). Treatment for acute anterior cruciate ligament tear: five year outcome of randomised trial. BMJ.

[7] Monk AP, Davies LJ, Hopewell S, Harris K, Beard DJ, Price AJ (2016). Surgical versus conservative interventions for treating anterior cruciate ligament injuries. Cochrane Database Syst Rev.

[8] Krause M, Freudenthaler F, Frosch KH, Achtnich A, Petersen W, Akoto R (2018). Operative versus conservative treatment of anterior cruciate ligament rupture. Dtsch Arztebl Int.

[9] Sandberg R, Balkfors B, Nilsson B, Westlin N (1987). Operative versus non-operative treatment of recent injuries to the ligaments of the knee. A prospective randomized study. J Bone Joint Surg Am.

[10] Markström JL, Tengman E, Häger CK (2018). ACL-reconstructed and ACL-deficient individuals show differentiated trunk, hip, and knee kinematics during vertical hops more than 20 years post-injury. Knee Surg Sports Traumatol Arthrosc.

[11] Filbay SR, Roos EM, Frobell RB, Roemer F, Ranstam J, Lohmander LS (2017). Delaying ACL reconstruction and treating with exercise therapy alone may alter prognostic factors for 5-year outcome: an exploratory analysis of the KANON trial. Br J Sports Med.

[12] Pinczewski LA, Lyman J, Salmon LJ, Russell VJ, Roe J, Linklater J (2007). A 10-year comparison of anterior cruciate ligament reconstructions with hamstring tendon and patellar tendon autograft: a controlled, prospective trial. Am J Sports Med.

[13] Buss DD, Min R, Skyhar M, Galinat B, Warren RF, Wickiewicz TL (1995). Nonoperative treatment of acute anterior cruciate ligament injuries in a selected group of patients. Am J Sports Med.

[14] Kumar S, Singh K, Chadha G (2018). Comparative study of anterior cruciate ligament reconstruction versus conservative treatment among non-athletes: a 10-years follow-up. J Orthop Trauma Surg Relat Res.

[15] Meuffels DE, Favejee MM, Vissers MM, Heijboer MP, Reijman M, Verhaar JA (2009). Ten year follow-up study comparing conservative versus operative treatment of anterior cruciate ligament ruptures. A matched-pair analysis of high level athletes. Br J Sports Med.

[16] Daniel DM, Stone ML, Dobson BE, Fithian DC, Rossman DJ, Kaufman KR (1994). Fate of the ACL-injured patient. A prospective outcome study. Am J Sports Med.

[17] Casteleyn PP, Handelberg F (1996). Non-operative management of anterior cruciate ligament injuries in the general population. J Bone Joint Surg Br.

[18] Maletius W, Messner K (1999). Eighteen- to twenty-four-year follow-up after complete rupture of the anterior cruciate ligament. Am J Sports Med.

